# A multi-site mixed-method evaluation of ‘Cardiac College for Women’ implementation: perspectives of cardiac rehabilitation patients and providers

**DOI:** 10.3389/fcvm.2024.1430268

**Published:** 2024-10-08

**Authors:** Rachael Pamela Carson, Sherry L. Grace, Ana Paula Delgado Bomtempo, Andree-Anne Hebert, Marie-Kristelle Ross, Paul Oh, Gabriela Ghisi

**Affiliations:** ^1^ Faculty of Health, York University, Toronto, ON, Canada; ^2^ KITE Research Institute, Toronto Rehabilitation Institute, University Health Network, University of Toronto, Toronto, ON, Canada; ^3^ Graduate Program in Physical Education, Faculty of Physical Education and Sports, Federal University of Juiz de Fora, Juiz de Fora, Brazil; ^4^ Programme de Prévention Secondaire et Réadaptation Cardiovasculaire, Levis, QC, Canada; ^5^ Department of Physical Therapy, Temerty Faculty of Medicine, University of Toronto, Toronto, ON, Canada

**Keywords:** cardiac rehabilitation, women's health, patient education as topic, patient compliance, patient satisfaction, health personnel

## Abstract

A *Cardiac College for Women* curriculum was developed to address the dearth of women-focused cardiac rehabilitation (CR) education. This study investigated: (1) patient utilization of the education; (2) acceptability and applicability of the education; as well as (3) patient and CR providers’ experiences implementing it. This was a multi-site, mixed-methods study. After baseline assessments at an academic CR program in two Canadian provinces, the 12 weekly 30 min structured in-person group education sessions were led by staff in the relevant discipline, with supporting online videos and written materials. Women reported their engagement with the education in weekly diaries, and completed a survey post-program. Semi-structured virtual interviews were held with willing participants and staff delivering the intervention. Transcripts were analyzed concurrently by two researchers independently via NVIVO using text condensation, followed by consensus reconciliation and multi-source validation. Forty patients participated in the women-focused education program, with 28 completing weekly diaries and 36 post-intervention surveys. Participants attended 80% of sessions (67%–89%). They spent an average of 30 min/week engaging additionally with the online education, with 83% rating the weekly content applicable (73%–100%). Overall acceptability was rated 4.3 ± 1.7/5. Twelve patients and 5 staff participated in interviews. Four themes were identified: contextual considerations, staffing and implementation issues, valued aspects, and suggestions for improvement. In conclusion, *Cardiac College for Women* was established as highly acceptable and applicable to patients, supporting their self-management. The women-specific CR education materials were also established as readily implementable by CR staff.

## 1 Introduction

Cardiovascular disease (CVD) is the leading cause of death worldwide (1), with 20% of individuals dying after an initial cardiac event (2). Due to advances in care improving survival, CVD is also one of the most prevalent conditions globally (3). Given that 40% of patients experience another cardiovascular event within three years (2, 4), prioritizing secondary prevention is crucial. Cardiac rehabilitation (CR) is an established outpatient model of comprehensive secondary prevention composed of risk factor modification, exercise training, nutrition counselling, psychosocial management, and notably, patient education (5). Patients are empowered through education to recognize and respond to cardiac symptoms, as well as to actively manage their cardiovascular risk factors, to reduce their risk of further sequelae (6). Robust evidence has demonstrated that CR reduces mortality and morbidity (5, 7), as well as improves functional capacity (8) and quality of life (9).

Despite its benefits, few women access CR (10, 11), and where they do, often they do not achieve the same gains as men (12). Reasons are multifactorial, and include transportation barriers, family obligations, as well as finding exercise to be painful or tiring, often due to their different forms of CVD or burden of comorbidities when compared to men (13). Resources to optimize secondary prevention for women are needed. Indeed, an international clinical practice guideline advocating for women-focused CR was recently published, recommending the tailoring of education to target women's needs (14).

Accordingly, our group rigorously developed *Cardiac College for Women*, which offers a comprehensive CR curriculum available open access in multiple formats (15). Preliminary evaluation confirmed its efficacy in improving cardiac knowledge and clinically-prognostic outcomes, such as functional capacity (16). Alongside the prospective evaluation, its implementation was also investigated. The objectives were to: (1) assess degree of utilization of *Cardiac College for Women* education materials throughout CR; (2) investigate women participants’ perceptions of the acceptability, applicability and usability of the tailored education and their satisfaction with it; and (3) explore CR provider's experiences adopting, implementing, and sustaining delivery of the education curriculum in their programs.

## 2 Materials and methods

### 2.1 Design

This was a multi-site, mixed-methods study (protocol registered at: https://osf.io/b2jz3/). Mixed-methods were applied to achieve contextualization, validation and triangulation of findings, enhance interpretation of findings, and achieve comprehensive understanding of the research questions. An exploratory, sequential design was used for this two-staged study (17): the first stage comprised a prospective, uncontrolled sub-study, and a subsequent qualitative study. Data were collected between September 2023 and March 2024.

Study approval was obtained from the University Health Network (Toronto, Canada: 23-5427) and Centre Intégré de Santé et the Services Sociaux de Claudiére-Appalaches (Lévis, Canada: 2024-1076). All participants provided written informed consent.

### 2.2 Intervention: women-focused CR education

The women-focused education intervention for this study—*Cardiac College for Women*—was rigorously developed following a user needs assessment, and considering health literacy as well as principles of adult education, as summarized elsewhere (15). It was delivered within a 12-week in-person women's-only CR class (schedule of topics shown in Table 1), with education and individually-prescribed supervised exercise at each session (16).

**Table 1:** Engagement with, as well as utilization and applicability of, weekly Cardiac College for Women content by mode (*n* = 28).

Weekly topic		In-person education session	Supportive online written materials	Supportive online videos	Optional website information	Applicability
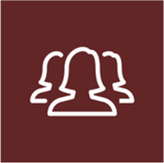	Orientation	19 (70%)	18 (67%) *3 (11%)*	20 (74%) *1 (4%)*	21 (81%)	20 (74%) *6 (22%)*
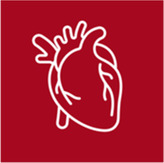	About your heart	22 (82%)	17 (63%) *0 (0%)*	19 (70%) *2 (7%)*	19 (73%)	22 (82%) *3 (11%)*
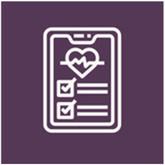	Cardiovascular risk factors	22 (82%)	18 (67%) *1 (4%)*	20 (74%) *3 (11%)*	18 (69%)	23 (85%) *3 (11%)*
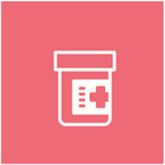	Cardiac medications	24 (83%)	19 (66%) *2 (7%)*	18 (64%) *4 (14%)*	21 (75%)	25 (89%) *1 (4%)*
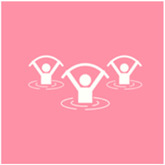	Exercise (aerobic)	22 (79%)	19 (73%) *3 (12%)*	23 (85%) *2 (7%)*	21 (81%)	26 (100%) *0 (0%)*
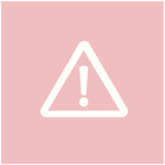	Exercise (safety)	22 (88%)	18 (72%) *3 (12%)*	18 (72%) *1 (4%)*	18 (72%)	21 (88%) *2 (8%)*
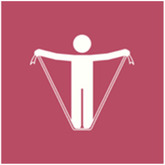	Exercise (resistance training)	21 (81%)	19 (73%) *1 (4%)*	17 (65%) *2 (8%)*	20 (77%)	25 (96%) *1 (4%)*
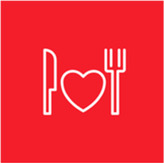	Nutrition (Mediterranean pattern)	23 (89%)	13 (54%) *2 (8%)*	19 (73%) *2 (8%)*	19 (73%)	19 (73%) *6 (23%)*
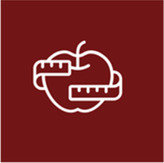	Nutrition (Healthy relationship with food)	18 (67%)	16 (64%) *2 (8%)*	21 (78%) *1 (4%)*	21 (78%)	20 (74%) *5 (19%)*
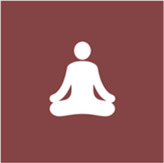	Psychosocial health (Depression and anxiety)	21 (81%)	15 (58%) *2 (8%)*	18 (69%) *0 (0%)*	18 (69%)	20 (77%) *1 (4%)*
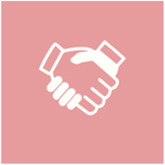	Psychosocial health (Relationships and self-management while juggling multiple roles)	18 (78%)	12 (52%) *4 (17%)*	19 (79%) *1 (4%)*	16 (70%)	19 (79%) *2 (8%)*
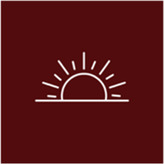	Maintenance post-program	19 (83%)	14 (64%) *1 (4%)*	18 (78%) *1 (4%)*	16 (76%)	16 (76%) *4 (17%)*
**Mean percentage yes across all sessions**		**80%**	**73%**	**80%**	**75%**	**83%**

Valid percentages reported. Non-italicized *n* (%) for yes responses. Italicized *n* (%) for “somewhat” responses (where queried).

The group education program comprised didactic sessions based on structured learning plans regarding CVD self-management and healthy behavior change (16). The slides guiding each session were similarly developed through a rigorous process as outlined above, and are available upon request (15). Each education session was planned for 30 min. Participants were also referred to specific corresponding supportive information on the *Cardiac College for Women* website (open access) (15), with over 70 assets available in text or video.

Compared to men on average, women often have different CV diagnoses (e.g., spontaneous coronary artery dissection [SCAD], myocardial infarction with non-obstructive coronary arteries [M/INOCA], cardiomyopathy), different risk factor burden (i.e., tobacco use, diabetes, peri/menopause), and comorbidities (i.e., alcohol or cannabis abuse, cancer, osteoporosis, autoimmune disease, polycystic ovarian syndrome); these may complicate their CVD management. While women require education about these and it is not readily available, they are not universal and hence it is not cost or time efficient or prudent (e.g., applicability, patient preference) for the CR education program to be exhaustive. Thus, as applicable, specific content about each of these clinical matters was offered to individual participants. Where agreed, relevant website links were provided via email; patients were directed to follow-up with their CR supervisors with any further questions.

### 2.3 Setting

This study was conducted in two Canadian CR programs: Toronto Rehabilitation Institute, University Health Network in Toronto, Ontario and Prévention Secondaire et Réadaptation Cardiovasculaire in Lévis, Quebec. They are both academic institutions.

Staff at both centers were provided the 12 education session slide sets in the applicable language (15). Training by the last author was provided for the relevant disciplines who would be delivering each session: 6 staff at the Ontario center and 4 at the Quebec center.

The CR programs including the education were offered in English at the Ontario site, and in French at the Quebec site (15). The education was delivered in a group setting (no rolling intakes) over a 12-week period, in-person, and led by a case-managing physiotherapist or kinesiologist for care continuity. Both programs were comprehensive and delivered based on clinical practice guidelines (18–20).

### 2.4 Participants

Patients identifying as women who presented at the CR centers during the period of recruitment were included in the study if they had one of the following conditions or procedures: acute coronary syndrome, revascularization (i.e., percutaneous coronary intervention or coronary artery bypass graft surgery), SCAD, M/INOCA, Takotsubo cardiomyopathy, or heart failure (with preserved or reduced ejection fraction; HFpEF). Women at the Toronto site were given the choice of program model in which to enroll based on their preferred class time, and only those in the women-only model were included in this sub-study. At the French site, all women enrolled in the women-only model during the period of study were invited to participate.

The inclusion criteria were age >18 years, as well as access to the internet and a computer, tablet and/or smartphone. The exclusion criteria were: any comorbid physical, sensory, or serious mental condition which would interfere with the patient's ability to read and participate in in-person education sessions, and lack of English or French-language proficiency. Patients who only had a smartphone (i.e., no access to larger screen) were excluded from being interviewed.

The inclusion criteria for CR staff were being involved in the delivery of the women-focused education materials at the centres under study. There were no exclusion criteria. For both patients and staff, interviews were held until thematic saturation was achieved (21).

### 2.5 Procedures

Baseline clinical data were extracted from medical charts. Assessment of participant sociodemographic characteristics was undertaken before CR started via online or paper versions (based on patient preference) of identical surveys.

Participants were similarly provided with a diary, and they were reminded to complete it each week of the education program. At 3 months (in accordance with the end of the CR programs and corresponding education), a survey was administered again either online or on paper.

After the intervention, patients in the intervention group at both sites were contacted by the research team via email and were invited to participate in one virtual interview. Similarly, staff who administered the education materials were emailed an interview invitation.

Semi-structured one-on-one interviews were conducted with willing participants via Zoom, with live transcription enabled and cameras on. The interviews were conducted by the first author (RC), who was trained by the last and second author (GG, SG); a second team member observed to record nonverbal communication. The first author is a master's student who underwent qualitative methods training and was not involved in the development or delivery of the education. She only knew the participants through study recruitment and data collection (which was often virtual). The interview guide was displayed on screen throughout.

### 2.6 Measures

To characterize the sample, patient clinical characteristics extracted included cardiovascular diagnoses/procedures, risk factors and comorbidities. Patients self-reported their sociodemographic characteristics, including gender-related factors (22).

The weekly diaries were used to track participant utilization of, engagement with, and their perceptions of the applicability of, the *Cardiac College for Women* intervention. Items were investigator generated, with forced-choice response options, such as whether they attended the session (yes or no; for utilization), whether they went to the website for more information (engagement), and whether the content was deemed relevant to their lives (yes, somewhat, or no; applicability). Each week's diary concluded with an open-ended item where participants were directed to add any questions or comments.

The utilization and acceptability surveys administered post-intervention pertained to the full 12 weeks of education. Utilization items were also investigator generated. Attendance at sessions, completion of weekly assigned readings and watching of assigned videos were assessed, using 5-point Likert type scales from 1 (never) to 5 (always), a question about amount of time spent (minutes), and proportion of assigned materials covered (%). Using similar response options, participants were also asked about their use of the website for additional learning. Participants were also asked about whether they agreed to receive additional individualized education for topics not covered in the curriculum; if so, they were asked about their degree of independent engagement with those online materials, and whether they contacted their CR provider(s) with queries about the content. Acceptability items –shown to be valid in previous work (23, 24)—were adapted for this curriculum, with 2 additional items created. The responses for the 14 items ranged from 1 (strongly disagree) to 5 (strongly agree) on a Likert-type scale. One item was reverse-scored to minimize acquiescence bias. An overall mean acceptability score was computed.

Two semi-structured interview guides were developed by SG and GG, one for patients and one for staff. They were informed by our previous literature review (16), and input from the investigative team. Questions and corresponding probes for patients explored overall satisfaction with content and delivery, how the progression of weekly topics flowed from session to session and degree to which it was perceived integrated, specific applicability and implementability into their lives, as well as suggestions for improvement. Questions and corresponding probes for patients explored implementability into the program, applicability, sustainability, and similarly suggestions for improvement. They were pilo*t*-tested before formal interview initiation, and refined accordingly. The auto-generated transcripts were cleaned verbatim, except to preserve anonymity.

### 2.7 Data analyses

Participant characteristics were analyzed using descriptive statistics (e.g., frequency and percentage for categorical data; mean and standard deviation for continuous data). Utilization and acceptability ratings were similarly analyzed. Open-ended diary responses were content analyzed manifestly (25).

The qualitative data were analyzed using an inductive and text-driven approach to ensure rigor (26). Given that two different stakeholder groups were involved, transcripts were analyzed using systematic text condensation (27). Comments related to CR in general (i.e., non-education components such as exercise, general non-women-focused education) were not the focus of analysis. NVivo software (version 14) was used for data organization and coding, concurrently with interviews (28).

After training and calibration, two researchers (RC, AB) initially read through the the patient and staff transcripts independently, to get an overall sense of the content and identify preliminary themes from the raw data. They then broke the data down into smaller units of meaning, which they sorted into code groups based on thematic similarities. The researchers then met to review the coding and condense it to distill the essence of the data, transforming the codes into more concise and meaningful summaries. This condensed data was brought forward to a third author (GG) to discuss and reconcile where needed. The coding was then synthesized into descriptions and concepts to provide a coherent narrative and deeper understanding of the themes identified. For member checking, themes and their corresponding subthemes were shared with the education staff from both programs. Subsequently, themes were shared with the second author (SG) for validation. Data saturation was considered, confirming that no new themes were identified. Finally, themes were shared with all authors, such that consensus was reached, ultimately ensuring credibility and trustworthiness of the findings (29).

The quantitative and qualitative methods were integrated by means of merging, such that results of both stages were then brought together for analysis contiguously. By comparing and cross-verifying the results from both methods, consistencies and discrepancies were identified, to increase the validity and reliability of conclusions. This methodological triangulation enabled a comprehensive understanding of *Cardiac College for Women* utilization, acceptability, applicability, usability and implementability from multiple perspectives. Interpretation of the integrated results was performed narratively, including consideration of fit of the results from the two stages (17), as well as with available literature.

## 3 Results

### 3.1 Participant characteristics

Of women approached during the period of study, 54.4% consented and met the inclusion criteria; 40 (19; 47.5% French-speaking) of these patients enrolled in the women-only program. Of these, 36 (90.0%) completed the post-intervention utilization and acceptability surveys and form the cohort for this study. Sociodemographic and clinical characteristics are shown in Table 2. Of these participants, 28 (77.7%) completed any of the 12 weekly diaries.

**Table 2:** Sociodemographic and clinical characteristics of patient participants.

Characteristic	Retained quantitative sample (*N* = 36)	Qualitative interviewees (*n* = 12)
Sociodemographic & gender-related characteristics		
Site (Toronto)	19 (52.8%)	5 (41.6%)
Age (years)	66.3 ± 9.7	67.8 ± 9.8
Ethnocultural background		
White/Western European	32 (91.4%)	12 (100.0%)
South Asian	1 (2.9%)	0 (0.0%)
Asian	1 (2.9%)	0 (0.0%)
Multi-racial or other	1 (2.9%)	0 (0.0%)
Highest education		
Some secondary school	8 (22.9%)	1 (8.3%)
Some college	13 (37.1%)	6 (50.0%)
Some university or greater	14 (40.0%)	5 (41.7%)
Health literacy^a^	17.8 ± 2.9	19.0 ± 1.4
Limited	1 (2.8%)	0 (0.0%)
Marginal	7 (19.4%)	0 (0.0%)
Adequate	28 (77.8%)	12 (100.0%)
Work status		
Paid employment (full or part-time)	13 (36.1%)	5 (41.7%)
Other (disability, retired, household management)	23 (63.9%)	7 (58.3%)
Financial worry^b^	3.2 ± 1.1	3.2 ± 0.9
Neighborhood environment walkable (e.g., treed, safe, low pollution)	32 (88.9%)	12 (100.0%)
Marital status		
Married or equivalent	21 (60.0%)	6 (50.0%)
Widowed or single	10 (28.6%)	3 (27.3%)
Divorced or separated	4 (11.4%)	2 (18.2%)
Number of children	1.7 ± 1.2	1.8 ± 1.0
Children reside with patient (% yes)	8 (22.9%)	2 (16.7%)
Living situation		
With partner and/or family	26 (72.2%)	8 (66.7%)
Alone	10 (27.8%)	4 (33.3%)
Emotional support^b^	4.3 ± 0.9	4.5 ± 0.8
Health support^b^	4.6 ± 0.7	4.6 ± 0.7
Informal caregiving responsibilities		
Yes	5 (13.9%)	0 (0.0%)
Sometimes	2 (5.6%)	1 (8.3%)
Hours/week	14.8 ± 8.4	10.0 ± 0.0
Most responsible for domestic management in home	26 (74.3%)	7 (58.3%)
Number of hours spent per week on housework	9.1 ± 7.5	8.7 ± 7.4
Clinical characteristics		
CR indication^c^		
Myocardial infarction	11 (30.6%)	5 (41.7%)
Coronary artery disease	11 (30.6%)	6 (50.0%)
PCI	9 (25.0%)	3 (25.0%)
Valve disease	2 (5.6%)	1 (8.3%)
CABG surgery	3 (8.3%)	0 (0.0%)
Stroke/TIA	4 (11.1%)	1 (8.3%)
SCAD	2 (5.6%)	0 (0.0%)
MINOCA/INOCA	2 (5.6%)	1 (8.3%)
HFrEF	2 (5.6%)	0 (0.0%)
Stable angina	1 (2.8%)	0 (0.0%)
Cardiomyopathy	2 (5.6%)	0 (0.0%)
Other (e.g., arrhythmia, atrial fibrillation, pacemaker, primary prevention)	23 (63.9%)	7 (58.3%)
CV risk factors^c^		
Dyslipidemia	26 (72.2%)	10 (83.3%)
Hypertension	19 (52.8%)	6 (50.0%)
Diabetes	6 (16.7%)	2 (16.7%)
Overweight (BMI > 25 kg/m^2^)	7 (19.4%)	1 (8.3%)
Depression and/or anxiety	8 (22.2%)	3 (25.0%)
Alcohol use (≥once/month)	8 (22.2%)	4 (33.3%)
Comorbidities^c^		
Chronic pain/joint repair or replacement	8 (22.9%)	5 (41.7%)
Autoimmune disease	5 (14.3%)	1 (8.3%)
Osteoporosis	5 (14.3%)	2 (16.7%)
Sleep apnea	6 (16.7%)	1 (8.3%)
Cancer history	6 (17.1%)	2 (16.7%)
Tobacco use (current)	1 (2.8%)	1 (8.3%)
COPD	1 (2.9%)	0 (0.0%)
Musculoskeletal issues	2 (5.6%)	2 (16.7%)
Osteoarthritis	1 (2.8%)	0 (0.0%)
Frailty	1 (2.8%)	1 (8.3%)

BMI, body mass index; CABG, coronary artery bypass graft; COPD, chronic obstructive pulmonary disease; CR, cardiac rehabilitation; CV, cardiovascular; HFrEF, heart failure with reduced ejection fraction; INOCA, ischemic and non-obstructive coronary arteries; MINOCA, myocardial infarction with non-obstructive coronary arteries; PCI, percutaneous coronary intervention; SCAD, spontaneous coronary artery dissection; TIA, transient ischemic attack.

*n* (valid%) or mean ± standard deviation shown.

^a^ Scores range from 4 to 20, with classifications of limited health literacy (4–12 points), marginal health literacy (13–16 points), and adequate health literacy (17–20 points).

^b^ Rated on a 5-point Likert-type scale, such that higher scores denote greater endorsement of the given construct.

^c^ As many as applicable reported.

### 3.2 Patient education use, engagement, acceptability and applicability

Weekly engagement with *Cardiac College for Women* elements as reported in the diaries is shown in Table 1; it was quite consistently high across all topics. Attendance at synchronous education sessions ranged from 67%–89% across all topics. Participants generally watched assigned weekly videos more often than read materials, with the notable exception of the resistance training content. Generally, at least 75% of the time participants visited the website for more information about a given topic, with the notable exception of the psychosocial content, and that about risk factors. Finally, with the exception as expected of the introduction and concluding sessions, relevance/applicability of the content to women's lives was rated very highly, other than the sessions regarding nutrition, and to a lesser degree psychosocial health.

Overall utilization of *Cardiac College for Women* materials across the program as shown in Table 3 echoes the weekly data. Participants almost always completed the weekly education. Participants read supporting weekly assigned readings or watched assigned videos at least “sometimes” or more, engaging with over two-thirds of this material for 30 min per week an average, be it reading or watching videos. Participants rarely perceived a need to contact CR staff with questions outside of the education sessions. Nine participants accepted the additional education based on reporting a non-universal CR indication, CV risk factor or comorbidity not covered in the curriculum, all of whom reported accessing the material provided. Correspondingly, Table 4 displays perceived curriculum acceptability, revealing high ratings by all participants (all means ≥4/5).

**Table 3:** Overall utilization of Cardiac College for Women educational resources (*N* = 36).

Item	Mean ± SD
How often completed recommended weekly heart education (/5^a^)	4.0 ± 0.9
How often read the assigned materials (/5^a^)	3.9 ± 1.0
Mean minutes per week reading the materials	30.3 ± 18.0
Proportion of weekly assigned reading completed (%)	71.7 ± 32.9
How often watched the assigned videos (/5^a^)	3.6 ± 1.3
Mean minutes per week watching the videos	30.1 ± 24.5
Proportion of weekly assigned videos watched (%)	63.9 ± 37.1
Accepted any additional educational material based on indication, risk factor or comorbidity not addressed in group education program^b^ (n,% yes)	9 (47.6%)
Additional education completed	9 (100.0%)
How often visited the Cardiac College website to learn more about any educational topic (/5^a^)	3.3 ± 1.1
Mean minutes per week learning over and above assigned content	28.6 ± 25.0
Contacted the healthcare team to ask questions about the educational content outside of the synchronous group education sessions (/5^a^)	1.7 ± 1.0

^a^ Scored from 1 “never” to 5 “always”.

^b^ e.g., diabetes, alcohol use, obesity, spontaneous coronary artery dissection, myocardial infarction with non-obstructive coronary arteries.

SD, standard deviation.

Valid percentages reported.

**Table 4:** Assessment of acceptability of Cardiac College for Women education materials^a^ (*N* = 36).

Item	Mean ± SD
1. The education met my approval	4.4 ± 0.9
2. The education was appealing to me	4.5 ± 0.8
3. I liked the education	4.5 ± 0.8
4. The education helped me improve my heart health	4.3 ± 0.9
5. I will use the information in my daily life	4.5 ± 0.8
6. I understood all the information	4.2 ± 0.8
7. The content was clear	4.4 ± 0.9
8. The information was relevant to my life	4.5 ± 0.9
9. The information was difficult to understand^b^	4.0 ± 0.1
10. I got all the information I was needing	4.2 ± 0.9
11. I will refer to this information again	4.4 ± 0.8
12. I would recommend the education to my friends and family	4.5 ± 0.8
13. The information was easy to read and hear	4.3 ± 0.9
14. The education was too long^b^	4.1 ± 1.3
**Overall Acceptability Rating**	**4.3** ± **1.7**

^a^ Acceptability scores ranged from 1 to 5, with 1 = strongly disagree to 5 = strongly agree.

^b^ Reverse-scoring shown, and included when computing the overall mean acceptability rating.

### 3.3 Qualitative perspectives of patients and programs

Of the 36 intervention-completing participants, 12 (33.3%) agreed to be, and subsequently were, interviewed. Sociodemographic and clinical characteristics of interview participants are shown in Table 2. While generalizability is not a goal of qualitative research, data in the Table suggests interview participants were generally representative of the program participants, although they may have been somewhat more educated and health literate, had fewer informal caregiving and domestic obligations (leaving them more time to be interviewed) and they may have suffered more comorbidities.

Five of the 10 involved staff consented to participate, and were interviewed; four were from the Toronto site. All were female. The following disciplines were represented: kinesiology (*n* = 2, 40.0%), dietetics (*n* = 2, 40.0%), medicine (*n* = 1, 20.0%).

**Figure 1:**
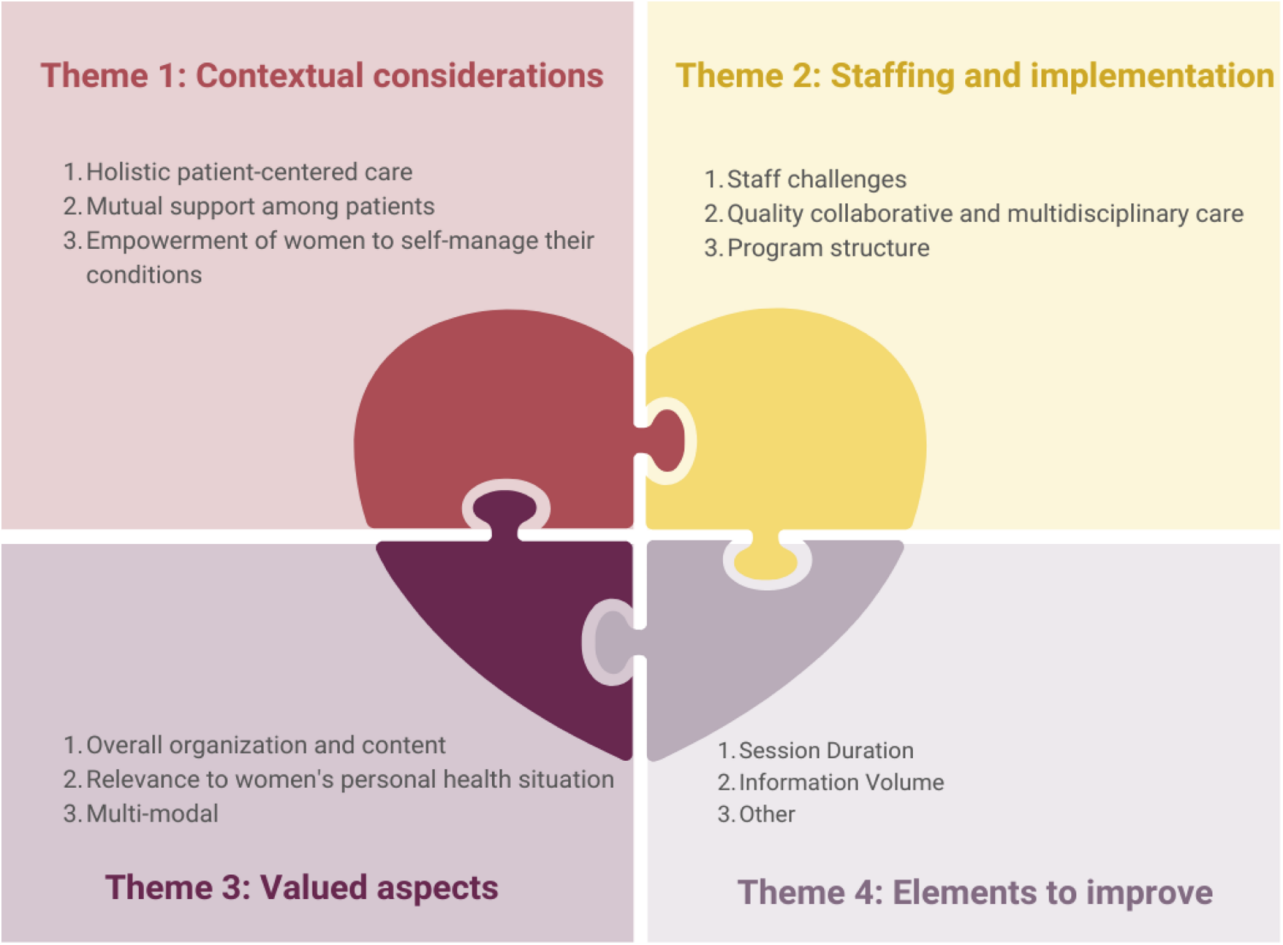
Themes and subthemes related to women-focused cardiac rehabilitation education and its’ implementation. CR, cardiac rehabilitation.

The 17 interviews conducted were of a median duration of 25 minutes (interquartile range: 17–34 min). Upon analysis of the interviews, it was agreed thematic saturation was attained (21). After excluding general comments about CR education (e.g., technological literacy of participants, hearing quality, desire for post-CR maintenance programming), four overarching themes were identified, with corresponding subthemes (Figure 1), each described below. Illustrative quotes supporting each are shown in Table 5.

**Table 5:** Illustrative quotes supporting themes and subthemes from patient and staff interviews regarding Cardiac College for Women.

Theme	Sub-theme	Illustrative quotes (participant #)
Contextual considerations for women-focused CR education	Holistic patient-centered care	1. “We were given lots of information. We were given time to absorb that information and make it part of our lived experience, so that it wasn't just information that we received for the mind alone.” (P02) 2. “I loved it. If it hadnt been for that, I could have searched via the Internet, but it wouldn't have been as complete.” (P09)
	Mutual support among patients	1. “I enjoyed being part of the group. I thought it was informative and it was kind of fun in a lot of ways. Just because it was nice to interact with other people who have, you know, maybe some similar issues.” (P05) 2. “Being in a group, it's motivating.” (P01) 3. “There were women with more intelligence and experience who retained [information] better than me. They helped me.” (P10)
	Empowerment of women to self-manage their conditions	1. “We were given courage, and you know, I could see that all around people. Other women were saying that, and you could see during the program how you could actually see a change in people.” (P02) 2. “I didn't want to get up this morning, but I know when I got here, the effort is worth it because I'm leaving here really happy.” (P10) 3. “It took me out of my comfort zone, and I fought my fear and now I'm going to the pool. You know I motivated myself, I motivated myself in my head, and then I knew I would finally do it.” (P07)
Staffing and implementation of women-focused CR education	Staff challenges	1. “The fact that some of the women you know, they had people picking them up or WheelTrans and things like that. They were very anxious about the time, so that would make me anxious about the [session] time.” (S01) 2. “The videos that were translated into French were really helpful. To make sure that women found the videos, I sent the links relating to the theme of the week directly by email; otherwise, they would have had difficulty.” (S07) 3. “Maybe there was a couple occasions I probably should have reviewed it [the weekly diary booklet] ahead of time.” (S01)
	Quality collaborative and multidisciplinary care	1. “For me what stands out is … the different professionals helping to create this content. So not only physicians but kinesiologists, dietitians and so on.” (S03) 2. “The fact that they were experts, yes, I thought that was great and I think they did a really good job in that they didn't talk down to people.” (P01) 3. “I would say the fact that they could all join together for the education was nice. I probably wouldn't grow the class size beyond eight in the class for one clinician.” (S01)
	Program structure	1. “I think it fit well with what we usually do at cardiac rehab.” (S04) 2. “[The] education component was done well. You felt that it flowed well and even just with the slides and things like that. It was easy to not only understand as a patient but easy to go through and deliver.” (S03) 3. “It is nice that it's not too big. I mean, it probably could be a little bigger than what we had because it started off being 10 people, but it didn't take long for a couple of people to walk out early on, and then it became the same five people every week.” (P05)
Valued aspects of women-focused CR education	Overall organization and content	1. “I do want to say that if that was the first one [women's-only class], it turned out to be pretty bang on. They did a really good job of setting something up.” (P05) 2. “The more I progressed in the program, the more interesting I found it.” (P07) 3. “When I went online and I saw pictures of the actual movement of the heart, it was so, so moving, you know, so the online material and the class material encouraged me to go further to what was available online.” (P02) 4. “Overall I found it very helpful and very informative.” (P01)
	Relevance to women's personal health situation	1. “As a layperson, I know generally how my heart works. But I mean, it really helped to understand a lot more about it because you don't get that information from your doctor.” (P05) 2. “I was delivering the “how your heart works” or something like this. Many women [were] saying that they … had no idea about the difference between men and women.” (S03) 3. “I liked being part of the women's group. I thought the information was very much more relevant and probably more aligned with me then I think the regular group from what I've heard.” (P03) 4. “Yes, those that resonated with me the most [are] those that I have written about the most in my weekly journal.” (P07) 5. “I think we were really able to deliver new information to just women, changing their life. That's what I've heard … from them.” (S03)
	Multi-modal	1. “I like the live presentations the best. It was nice to have someone there to describe or, you know, directly answer questions. You know, the videos are great to go back to.” (P05) 2. “I think it's good to leave two methods, because it's certainly not just me who love it the same. Two methods, because if this program was just synchronous, I don't think there would be as many participants.” (P07) 3. “If I missed any live news, I could go see it again later. Like a movie that you missed a bit and you can rewind. It went well. It adds to what you might have missed.” (P09) 4. “I think for some people … some of its online. Especially for people who don't have the time and don't have the type of job where they can take that kind of time off.” (P03) 5. “I made sure that I either scheduled the individual or the online group sessions and I made sure I read the material and watched the videos on Cardiac College.” (P01)
Elements to improve	Session duration	1. “You might want to consider lengthening just to allow people the opportunity to ask questions.” (P01) 2. “But just time wise, we didn't have the time. So, I know with the way the cardiac groups are sort of set up, there's only half an hour allocated for education. But maybe there's a way around it that we could do a 45 min session, so maybe half an hour talk with at least 15 min at the end.” (S02) 3. “But I would say to have 45 min for education and Q&A.” (S01)
	Information volume	1. “If there was an additional one or two weeks in order to spread out a couple of those topics so that you're not overwhelmed.” (P05) 2. “Another talk about psychosocial health….” (P06) 3. “But some topics in that session could be extended or even broken into two classes. It's really a lot in terms of content because we talk about anatomy and then just about symptoms, if I'm not mistaken. So, we could even break in two.” (S03)
	Other	1. “I found it more difficult at first because I didn't think it was instinctive to find the resources on the Cardiac College for Women [website]. So, it was difficult to make this clear to the women participating.” (S05)

CR, cardiac rehabilitation; P, patient; S, staff.

The first theme pertained contextual considerations for optimal women-focused CR education delivery, with 3 elucidatory subthemes. Firstly, a holistic, patient-centered context was raised as foundational for the education, entailing personalized care respective of participant's individual preferences and values. Secondly, mutual support among peers was considered as a foundational springboard, enabling participants to learn from others’ experiences, within an environment of respect and laughter. Finally, women discussed how the education empowered them to self-manage their health, through facilitation of action planning to achieve behavior change. The women-focused context and education enhanced their motivation and adherence to the program.

The second theme pertained to staffing and implementation, again with 3 subthemes. The first concerned staff challenges, such a lack of formal training in women-focused curricula content and limited time in their workday for session preparation for first-time delivery. The second subtheme concerned the provision of quality collaborative and multidisciplinary care. Participants duly recognized the efforts of diverse healthcare professionals in creating a comprehensive program. Despite a multitude of expert presenters, participants perceived the education as cohesive, based on the adeptness of a main CR lead. Lastly was structural considerations of the program, such as optimal class size. Relatedly, optimal delivery of some content was considered more suited to group vs. individual delivery; while the group dynamic was considered foundational as above, for some content individualized delivery was preferred (e.g., nutrition, psychosocial).

The third theme raised valued aspects of the women-focused CR education, which should be maintained and replicated where possible for optimal implementation. Firstly, participants appreciated the overall organization and content of the session topics, reporting the information provided as high-quality, comprehensive, and concise. They valued the cohesive integration and flow of educational material, prefaced by a helpful orientation. Women considered it convenient to access all materials through the *Cardiac College for Women* online platform, and the information was trusted. Secondly, that the content was relevant to women's unique personal health situations was highly valued. They previously did not have such in-depth yet tailored information about their diverse health conditions, risk factors, and backgrounds. Lastly, participants valued the multi-modal learning formats for their flexibility and convenience, given the CR barriers they experienced in their lives.

The last theme captured some ways to potentially improve the women-focused education. Participants expressed satisfaction with the volume of information provided in synchronous education sessions, yet desired more time for raising questions and sharing among participants. Some staff and patients suggested extending session duration from 30–45 min to avoid feeling rushed, with the additional time dedicated to such interactions to enhance the learning experience. In addition, they suggested considering additional weeks or sessions to extend the program duration, allowing for more comprehensive coverage of topics, and again to avert any rushing through content.

### 3.4 Merging of mixed-methods

While overall satisfaction, usability, acceptability, and applicability were quantitatively rated highly, some anomalies and discrepancies were elucidated in the qualitative findings. For instance, in the interviews, feedback regarding the nutrition education was that this information was highly desirable, but was preferred in an individual rather than group format. Indeed, for some topics, patients did not want generic education, but tailored information to their needs one-on-one. Other participants desired that the kinesiologist lead most group education sessions for continuity. Overall, through merged analysis, vagaries in individual preferences were elucidated (e.g., some patients had high health literacy or prior knowledge on a specific topic so the content a specific week was considered too simple, some patients preferred not to learn from the website but in-person, and vice versa), to inform future program implementation.

## 4 Discussion

The population of women with CVD is increasing globally, with a corresponding recognition of their unique risk and hence needs; sadly, these often go unaddressed. Recent CR guidelines call attention to this gap (14), stressing the need for women-focused education such as *Cardiac College for Women*. In this first implementation evaluation in programs and with patients, usability has been tested through a mixed-method study. Engagement was high, with 80% of the 12 sessions attended on average, and women doing a further 30 min per week in independent learning online. The education was liked, and deemed informative and relevant to women's lives. CVD patients reported implementing heart-health behavior changes based on the education provided. In light of evidence also supporting clinical efficacy of *Cardiac College for Women* (18), CR staff recommend the education curricula for other programs, where delivery staff and infrastructure are available.

Rigorous reviews have established that patient education may reduce morbidity, and significantly improves quality of life (30) and self-management in heart patients (31). However, there have been few studies regarding acceptability and implementation of patient education interventions in CR, or in other chronic diseases more broadly (32). Results herein are consistent with the many studies where the generic (i.e., non-women-focused) Cardiac College has been successfully adapted and implemented in diverse cultural contexts (33, 34). Barriers reported by staff related to lack of time and training in this study are common to those reported by patient educators for other chronic diseases (35).

### 4.1 Implications

Based on the results of this study, some revisions have been made to the education curricula. The generic (i.e., non-women-focused) education content on the *Cardiac College* website—such as regarding cardiovascular medications, among other clinical information—has been updated for currency. Navigation design could be improved so women can more readily find the specific information they seek. The team hopes to create a dedicated section on the *Cardiac College* website for professionals, to support programs and clinician providers implement the curriculum.

From a program perspective, some implementation matters merit consideration (36). First, program leadership should allocate sufficient time for staff to learn the needed content and prepare for optimal delivery, so they are adequately equipped to deliver the curriculum effectively. The Canadian Women's Heart Health Alliance offers clinician education for women and CVD which could fill staff education gaps (https://www.cwhha.ca/healthcare-professional-e-course-toolkit).

With regard to delivery format, while women desired and valued the group sessions, women-focused CR programs may better meet the needs of participants with individualized education regarding patient-specific clinical and psychosocial matters. To individually tailor education for less common CR indications and risk factors, women were directed to specific online educational resources to review independently in this study; this could ideally be done one-on-one with staff where resources permit. Local needs assessment regarding feasibility, staff expertise and patient preference for learning in each content area would facilitate optimal translation to other settings.

In addition, there were suggestions to extend the duration of each session and/or relatedly to increase the total number of sessions. Considering CR in Canada is among the longest globally, and the “dose” in terms of education time per program comparatively high (37), this likely cannot be heeded in most settings. *Cardiac College for Women* is multi-modal, such that where in-person synchronous congregation of women is infeasible, the online and asynchronous assets available freely online could be exploited to balance the preferences and needs of women with their lived realities and available program resources. Again, local needs assessment with patients and providers should be undertaken to optimally tailor the women-focused education.

In conjunction with supportive evaluation results (18), the curriculum should be tried in more diverse settings, with evaluation (36). Indeed, the open-access materials in English and French have been broadly disseminated through the International Council of Cardiovascular Prevention and Rehabilitation (ICCPR) community.

### 4.2 Limitations

Caution is warranted when interpreting these results. Foremost, generalizability is limited given that the study was only undertaken at two centres in a high-income country (although in one province CR is reimbursed by government health insurance and the other it is not). It is also limited given the small sample size, and the exclusion of participants with comorbidities. However, generalizability is not an aim of mixed-methods research. Second, results may be biased because the characteristics of patients who were eligible but not referred or were referred and did not enroll in CR are not available to compare to the characteristics of patients consenting to participate in this study. Thus, selection bias may be a concern, however retention bias is not.

With regard to design, the control participants from the larger quantitative study (18) were not asked to complete weekly diaries or complete post-program utilization and acceptability surveys. Thus, it is possible acceptability and engagement were as high with usual care, or that socially-desirable responding was at play. Indeed, the usability items in particular were investigator-generated hence with unknown reliability and validity. Future randomized, controlled research with objective measures of usability (e.g., website traffic) is warranted so causal conclusions may be drawn (36).

In conclusion, *Cardiac College for Women* has been demonstrated to be highly usable and used, as well as acceptable and applicable to the lives of CVD patients identifying as women. The education supported women's implementation of recommended disease management behaviors in their lives. Moreover, program staff perceived that the curricula and associated materials was highly feasible to implement, and reported that delivery can be sustained within their programs for the benefit of future women participants. Along with preliminary data supporting the efficacy of this education (18), overall results support dissemination to scale.

## Data Availability

The raw data supporting the conclusions of this article will be made available by the authors, without undue reservation.
